# Comminution of Dry Lignocellulosic Biomass, a Review: Part I. From Fundamental Mechanisms to Milling Behaviour

**DOI:** 10.3390/bioengineering5020041

**Published:** 2018-06-02

**Authors:** Claire Mayer-Laigle, Nicolas Blanc, Rova Karine Rajaonarivony, Xavier Rouau

**Affiliations:** UMR Ingénierie des Agropolymères et des Technologies Emergentes (IATE), University of Montpellier, CIRAD, INRA, Montpellier SupAgro, Montpellier, France; nicolas.blanc@inra.fr (N.B.); karine.rajaonarivony@supagro.fr (R.K.R.); xavier.rouau@inra.fr (X.R.)

**Keywords:** plant materials, mechanical stresses, energy consumption, grinding law, grinding

## Abstract

The comminution of lignocellulosic biomass is a key operation for many applications as bio-based materials, bio-energy or green chemistry. The grinder used can have a significant impact on the properties of the ground powders, of those of the end-products and on the energy consumption. Since several years, the milling of lignocellulosic biomass has been the subject of numerous studies most often focused on specific materials and/or applications but there is still a lack of generic knowledge about the relation between the histological structure of the raw materials, the milling technologies and the physical and chemical properties of the powders. This review aims to point out the main process parameters and plant raw material properties that influence the milling operation and their consequences on the properties of ground powders and on the energy consumption during the comminution.

## 1. Introduction

The use of biomass as a source of renewable energy, bio-based materials or green chemistry reagents has increased in recent years. In particular, the interest for lignocellulosic biomass has been growing as it presents the advantage for having a short production cycle while sequestering carbon. Lignocellulosic biomass produced annually by photosynthesis, represents an enormous feedstock estimated at approximately 79 GTOE/year (gigatons of oil equivalent, equivalent to about 320 Gtons/year) [1] while the annual world energy demand is about 12 GTOE/year (+2.5%/year). Lignocellulosic feedstocks occur as different types of raw materials which differ in compositions, properties, accessibility, processability. They can be categorized in different families: the agriculture and food processing by-products (straws, canes, stems, cobs, husks, hulls, brans, etc.), the dedicated energy crops (e.g., switchgrass, miscanthus, sorgho, etc.), the short and very short rotation coppices (e.g., willow, poplar, eucalyptus, etc.), the wood chain by-products (chips, sawdust, residues from felling and from second processing, etc.), the industrial and municipal wastes (residues of pallets, casing and packaging, green wastes, etc.) [2]. Whatever its origin, the input biomass needs to be calibrated to the transformation processes. The targeted size is function of the intended applications and can vary from several centimeters to a few micrometers. For example, in energetic applications, coarse particles in the range of few millimeters are targeted for anaerobic digestion processes [3] particles around 100 to 500 μm for biofuel production [4], and below 100 μm for gasification process [5] or direct combustion in engines [6,7]. Most of the time, in green chemistry applications, it is preferable to reduce the particle size below the cellular scale (~100 μm) in order to facilitate the extraction of molecules of interest [8,9]. When biomass is used as filler in bio-based materials, different particles properties could be sought by the comminution step. In case of composites intended for automotive equipment application, a significant aspect ratio is often sought to guarantee a good load transfer between the fiber and the polymeric matrix [10]. However in some applications of food packaging, it is preferable to decrease significantly the particle size (below 20 μm) in order to increase the proportion of filler content and reinforce the permeability properties [11]. In many cases, milling is still today seen as a common operation of particle size reduction for the pre-treatment of the biomass, and it is poorly studied in the scientific literature [12]. However, milling and especially fine milling can significantly influence the chemical and physical properties of the biomass and can be a lever to reveal some specific potentialities of the raw materials.

Based on a review of research works, this paper aims to highlight the major mechanisms occurring during the comminution of the biomass materials at the particle and process scales, their consequences on the properties of the ground powders and on the energy consumption.

## 2. Lignocellulosic Biomass Structure and Composition

Whole plants or their parts and primary products are constituted of different organs (trunks, stems, branches, leaves, roots…) ranging from several meters to few millimeters. At the histological scale (a few hundreds of μm) are the plant tissues made of clusters of cells with common natures and functions (vessels, parenchymae, supporting tissues, epidermis…). The cell sizes generally range from 100 to 10 μm depending on species and tissues. Plant cells are surrounded by a 0.1-to-several-μm-thick cell-wall and are generally devoid of cell content in most common lignocellulosic feedstocks. Lignocellulosic biomass comes mostly from secondary plant cell-walls [13]. The plant cell-wall is typically composed of cellulose (~20–60% dry weight basis, depending on biomasses), hemicelluloses (~10–40%) and lignin (~10–40%), with some associated minor constituents such as organic extractable compounds and mineral inclusions, for example silica deposits in rice husks [14].

The main component of the cell-walls, the cellulose, is a strictly water-insoluble assembly of linear polymers of beta-1-4-linked-d-gucopyranose units arranged in microfibrils containing both organized crystalline domains and amorphous regions. The degree of crystallinity refers to the ratio of crystalline to amorphous parts of cellulose [15]. Microfibrils are associated in cell-walls to form fibrils that can reach several μm length [16]. Hemicelluloses are a family of branched polysaccharides which form an amorphous matrix surrounding cellulose fibrils. They exhibit a great deal of compositional and structural variability depending on taxonomic classes, species and parts [17]. In the more common biomass feedstocks, main hemicellulose families are of glucurono-arabinoxylan type in grass plants and of galacto-glucomannantype and acetylated/methylated glucuronoxylan type in woods. Lignin is a tridimensional array of phenolic polymers resulting from the radical polymerisation of phenylpropenoid units. Its network impregnates secondarily the polysaccharide components of cell-walls and establish covalent linkages with hemicelluloses [18]. Lignin confers improved properties to the plant organs such as mechanical resistance, hydrophobicity, microbial protection, etc.

As an illustration, the composition of some biomasses is reported in Table 1 to give an idea of the variation range. The composition can vary significantly with the geographical area, the species and the analytical method thus, the data given here are indicative. Wheat straw and wood (pine), miscanthus and eucalyptus have been chosen to illustrate agriculture by-products, wood by-products, energy crops and short rotation coppices, respectively. The composition of industrial and agricultural wastes has not been reported as they highly depend on the origin and the formulation of the blend. Alfa is a perennial Mediterranean plant used for paper manufacturing, thanks to its high cellulose content. It is relatively close to wood pine, except for the lignin content which remains lower (12% against 25–30% for pine wood) and almost similar to miscanthus.

In addition, tissues from different parts of the plant can show different compositions. It is the case for example of flax and hemp fibers which are the external tissue of stem and present higher cellulose content (close to 80%) than flax shives and hemps hurds (core of the stalk). For full detailed compositions and other biomasses, reader can refer to the work of Vassilev et al. [19].

From a mechanical point of view, the structural organization of plant materials results in a fibrous material with an anisotropic orientation that exhibits different mechanical properties (Young’s modulus, tensile strength, Poisson coefficient, etc.) in the longitudinal and transverse directions [36].

**Figure 1:**
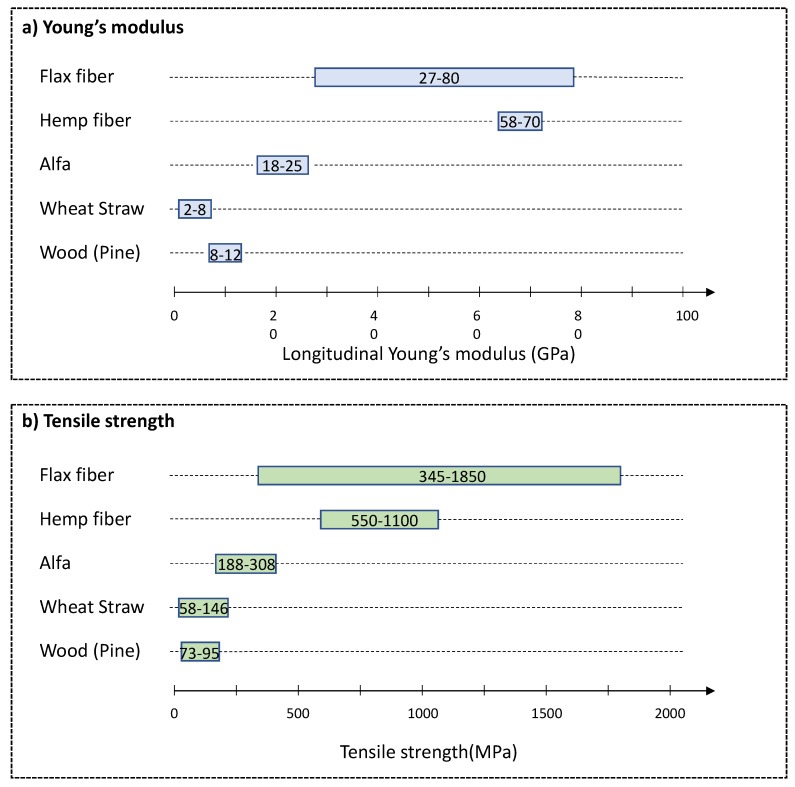
Main mechanical properties for different biomasses: (**a**) longitudinal Young’s modulus and (**b**) tensile strength according to [37,42].

As example, Figure 1 gives the range of the longitudinal Young’s modulus (a) and tensile strength (b) for different biomasses. The Young’s modulus or elastic modulus, is a measure of the stiffness of a material. It describes the elastic properties and the deformation behavior of the material. The tensile strength is the maximum stress that can be applied to a material before it breaks.

In Figure 1, we can note that these two properties are related partly to the cellulose content. Biomass with higher cellulose content and with cellulose microfibrils more aligned in the fiber direction (flax and hemp fibers) present higher mechanical performances in this direction [37]. However, the mechanical properties are also strongly influenced by the lignin content which depends on the growing and harvesting conditions, the plant maturity and the position in the stalk. In particular, Zhang et al. highlighted that a higher lignin content increases the Young’s modulus and decreases the tensile strength and elongation of single wood fibers [38].

Some fragilities could also be induced by heat or hydric stresses, failure during growing or water resorption during drying process. As biomass is a living material, it evolves with time under the action of humidity, temperature and decomposition modifying its mechanical properties [39]. As a consequence, a same biomass specie could present a high variability in properties which explains the large range for the mechanical properties in Figure 1.

In many studies, the mechanical properties of the biomass are determined in tensile tests. However, to fully characterize a biomass in view of its comminution, traction data in both directions, compression and shear are necessary. Indeed, the fibrous nature of materials, requires most of the time the use of shear during the comminution to reduce the length of the fibers. Several authors have adapted three-points bending tests for wheat straw and corn stalk pith in order to determine their mechanical properties under shearing stress [40,41]. In many case, these data are not available in the literature and are difficult to measure on biomass samples due to the high variability of the materials. In addition, in a grinder or in a mill, the mechanical stresses are applied randomly in the longitudinal and transverse directions and it is still very difficult to predict accurately the behavior of a specific biomass in the machines in relation to its mechanical properties.

## 3. Milling Processes

**Figure 2:**
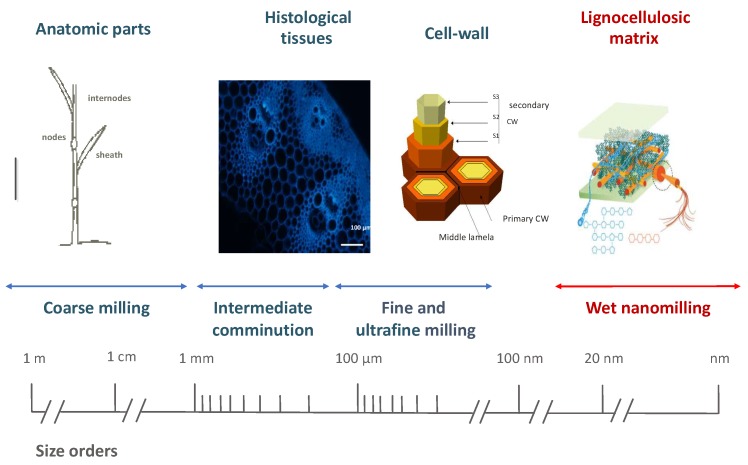
The metric and histological scales of a plant material in relation to the different milling steps.

Size reduction could be defined on the basis of an absolute scale associated to a metric size or an histological scale related to the structure of the plant materials as illustrated in Figure 2 [43]. Due to the heterogeneous structure of lignocellulosic biomass, the comminution involves generally several steps. The first one is generally coarse milling which leads to organ dissociation (from meter to centimeter) with cutting or crushing processes, then intermediate comminution (from cm to 1 mm) at the tissue scale, with shearing or impact processes, then fine milling at the tissue scale between 50 and 500 μm and finally ultra-fine milling, below the cell scale (<20 μm). Currently, it is not possible to reach particle size below 1 μm by dry milling processes. Thus the dissociation at the scale of the lignocellulosic matrix can be reached only by wet milling which is not in the scope of this review.

**Figure 3:**
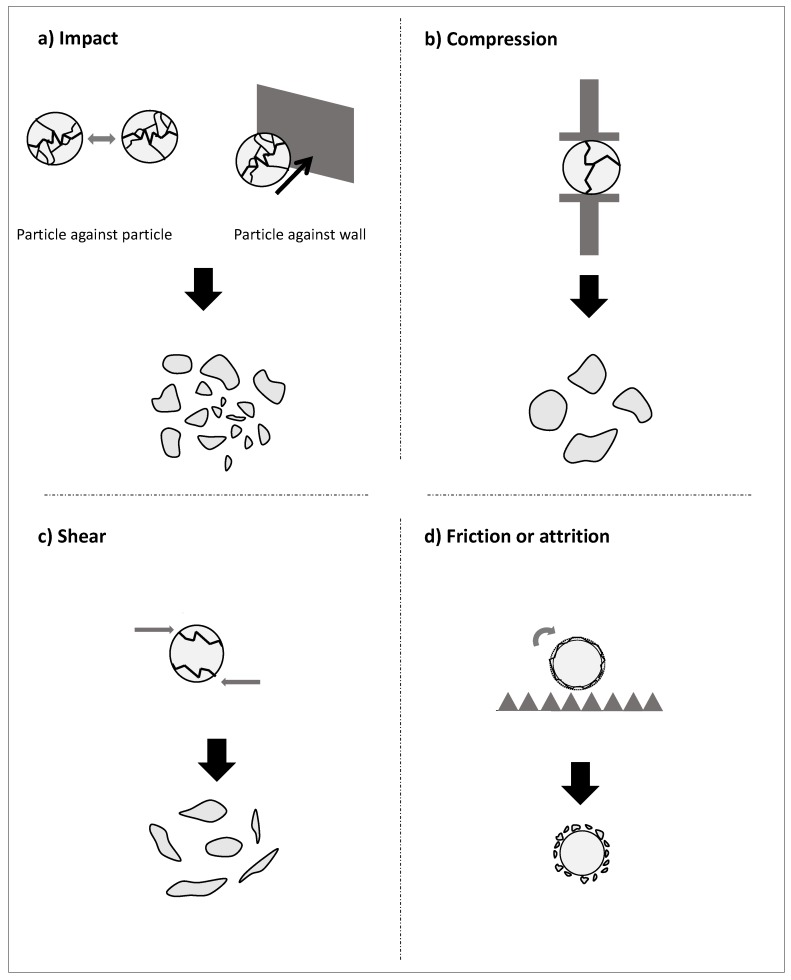
The main mechanical stresses in a grinder: (**a**) impact, (**b**) compression, (**c**) shear, and (**d**) friction and the particles that can be expected in case of comminution of an homogeneous materials according to Kaya et al. [45].

In practice, at the process scale, the different milling devices can be compared on the basis of the main mechanical stress they generate. Among them we can distinguish: compression, impact, shearing and abrasion/attrition, illustrated schematically in Figure 3.

The two first mechanical stresses (impact and compression) are similar in the force applied to the materials but in the first case, the energy is given to the materials in a quite instantaneous manner whereas in the second case the energy is transferred during a longer contact time. Impact and compression can be generated by projection of milling media (balls, bars in ball-mills) on particles, particles against one another (as in jet-mills for example) or against a wall or a milling tool (as in hammer-mills).

Shear and attrition act both also in quite a similar way on the materials. However, attrition is a surface mechanism, comparable to erosion of the materials and is generated by friction against walls, other particles or beads. Shear acts rather in the bulk of the materials and is often created by rolling mechanisms, and/or differential velocities between two milling tools.

## 4. Interactions between Plant Material and Mechanical Stress during the Comminution

**Figure 4:**
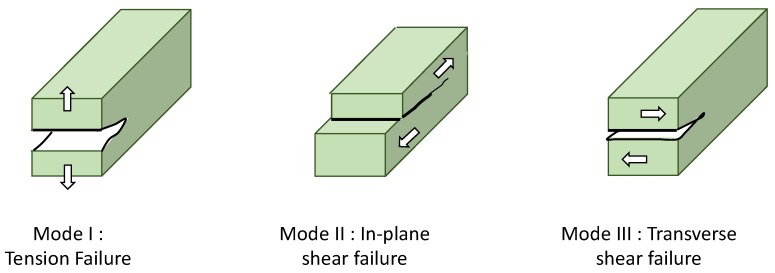
The different loading modes applied at the micro-scale leading to the failure of the materials according to Karinkanta [44].

The mechanical constraints (stresses) generated by the grinder are applied to the plant materials during the comminution process. The constraints need to be sufficient in order to overcome the failure strength (σ) and lead to the rupture of the materials. Indeed, if the applied force is weaker (under the yield stress), the material will deform under the load and will recover its initial shape when the load will be removed (this is known as elastic deformation). A greater load but under the failure strength will conduct to irreversible deformation (plastic deformation) of the material but without failure. Finally a load greater than σ will conduct to failure which will propagate inside the material according to its histological structure and the presence of internal defects. Three loading modes (see Figure 4) that conduct to different failure behavior can be broadly distinguished, one in tension (mode I) and two in shear (mode II and III). In the mode I, a tensile stress is applied and conduct to tension failure. The mode II and mode III lead to in-plane (forward) and out of plane (transverse) shear failures, respectively [44]. 

In a simplified approach, impact and compression could be associated to the loading mode I (traction) whereas shear and attrition to the loading mode II and III. In reality, at the scale of the particles, the mechanical loading is very complex. Indeed, in practice, the mechanical loading is applied to a more or less dense pack of particles and the local constraint on one particle is practically impossible to estimate. In addition, several mechanisms occur at the same time in the grinder but with various ratio of intensity according to the device and the process parameters [46]. For example, a rotation of the impactor during a compression leads to shearing action. Similarly, during shearing or attrition, the histological structure of the plant materials and the presence of internal defects could enable fracture in traction (mode I).

Whatever the way the breakage is initiated at the micro-scale, the fracture paths can propagate in different ways through the biomass. Its propagation depends on various process parameters (milling energy, mechanical stress, mechanical loading at the scale of the particle, etc.), properties of plant materials (histological structure, defects in the microstructure, mechanical properties, etc.) and environmental conditions (temperature, relative humidity, etc.) [47].

**Figure 5:**
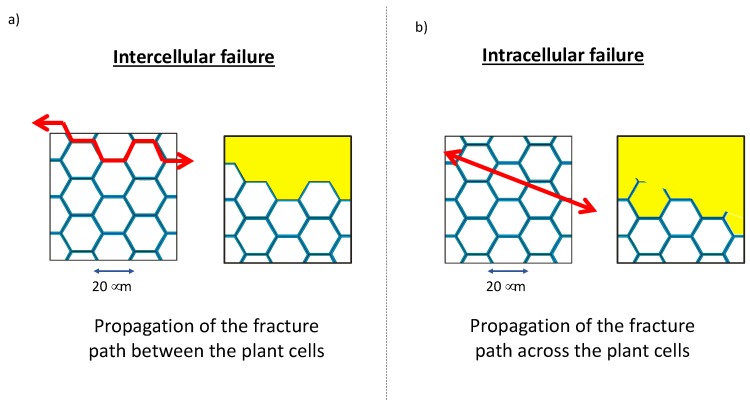
Intercellular failure (**a**) and Intracellular failure (**b**) observed during the milling of cork with impact and shear as main mechanical stresses, respectively [50]. (Tissue scale).

Two types of failures schematically represented in Figure 5 can be observed: (i) intercellular failure when the fracture path propagates between the cells leading to separation of the cells from each other (peeling) or (ii) intracellular failure when the breakage occurs across cells, disrupting secondary cell walls or trans-wall failure when the fracture path intersects the cell wall [48,49]. As an example, Motte et al. (2017) have shown that shear and compression generated by a millstone during the milling of cork induce intracellular failure (Figure 5b) whereas impact tends to generate intercellular failure (Figure 5a) [50].

In addition, certain mechanical stresses can be more efficient than others according to mechanical properties of the plant materials. A study on milling of Douglas fir bark in view of histological dissociation has highlighted that a visco-elastic plant tissue, such as the suber, deforms under impact and compression (in a ball-mill) but recovers its initial shape immediately after. For this tissue, this kind of loading engenders a high energy expenditure for a weak particle size reduction. In contrast, the phloem which is a more brittle tissue results in very fine particles under the same mechanical stress [9]. In a same way, a study on olive-pomace milling (composed of pulp and fruit kernel) has shown that attrition regime in a ball-mill allows a very fast particle size reduction of the pulp (friable) but has only a very low effect on the kernel which is a harder material [51].

## 5. Influence of Milling on the Properties of Particles (Size, Shape, Surface Area)

The main property of ground biomass powders affected by grinding processes is the particle size distribution. Several factors can be used to summarize the whole distribution but the most commonly used are the median size (*d*50), the percentiles 10th (*d*10) and 90th (*d*90) related to the proportion of fine and coarse particles in the powder, and the span which reflects the width of the distribution.

$$SPAN=\frac{d\left(90\right)-d\left(10\right)}{d\left(50\right)}$$

This size distribution is influenced by the grinder type and the process parameters (residence time, velocity of the grinder tools, etc.) [12]. Depending on the mechanical stress, particles will break in different ways as illustrated in Figure 3 [45,52]. Abrasion has tendency to create two types of populations by erosion of the raw first particles: very fine particles and coarser particles resulting in a median average size close to the original particle size. Compression tends to result in particles of similar diameters with a tighten particle size distribution. Impact, by explosion due to the transmission of a high energy in a very short time, will conduct to a wide range of particle sizes.

The structure of the plant materials plays also a key role on the resulting particle size distribution obtained from ground biomass. Most of the time, the different populations obtained are related to the histology and their relative importance are function of the mechanical stresses as illustrated in the work of Mayer-Laigle et al. (2017) on the milling of oat bran in different devices. The authors have shown that four populations (≤1 μm, ≈20 μm, ≈100 μm, ≈500 μm) appear during the milling steps. However, shear generated by the high shear mill and the pin-mill is more efficient to grind the material at the cellular scale, as these devices favor the particle population around ≈20 μm [47].

The morphology of the particles may also change during the process and can have an effect on the properties of the end-product as for the combustion of densified biomasses [12]. Although there are still few data on particle shape evolution during grinding, largely due to the difficulties to obtain enough representative and quantitative measurements especially for fine particles, several authors have shown that the shape of the particles obtained after milling is related both to the milling technology, the type of biomass and its history and moisture content [12,53]. The shape seems also related to the size of the ground particles. For rather homogeneous materials like minerals, it has been demonstrated that the smaller the particles, the more they tends to a regular shape that can be assimilated to a sphere or a parallelepiped rectangle [16,45,54]). The same tendency has been also observed, to a lesser extent for wheat straw [16] and Douglas fir wood [55] although the heterogeneity of the raw materials is greater. This can be related to the decrease in anisotropy of the biomass and the diminution of the number of defects as the size of the particles decreases.

The modification of the particle size and shape has a direct influence on the bulk density and the rheological properties of the powder [55,56]. Bulk density takes into account the pores and the inter-particular voids. A densification of the powder is observed during the milling both by the suppression of porosity and the reduction of inter-particular gap due to the creation of fine particles which fill the voids between coarse particles [57]. However, an inverse evolution of the bulk density can be observed in case of ultrafine milling. Indeed, new interparticular voids can be created by bridges between particles due to the Van der Waals forces [58]. The particle size reduction leads to an increase of the balance between the interparticular forces and the gravity force, intensifying the cohesion and decreasing the flowability. This has a direct impact on the handling and processing of fine powders [59]. This is particularly true for biomass powders which have a low density and are highly sensitive to humidity, in contrast with mineral powders. The particle shapes also highly affect the flowability as illustrated by Tannous et al. on ground Douglas fir wood powder. In their study, they show that particle shapes have a more significant effect on flow properties than particle sizes [55]. In a similar way, Chen and Qu and Zhao et al. have shown, by measuring the angle of repose on red rice and ginger powders that superfine powders have a lower angle of repose than coarser ones, which can be related to better flowability [60,61]. They explain this observation by the formation of aggregates with regular shapes in the fine powders.

The specific surface area, related to the particle size distribution, increases during grinding processes [62]. In addition, ultrafine grinding, by opening cell-walls modify the surface aspect of the particles making accessible some constituents that were trapped inside the cell. This leads to an increase of extraction yield for components of interest as illustrated by Hemery et al. on wheat bran [8]. In addition, comminution allows the rupture of the polysaccharide chains bonds and may increase their exposure at the surface of the particles. The result is an improvement of the enzymatic hydrolysis and an increase of the conversion rate of the biomass into bioenergy in bioethanol production [4,63]. This increase is also due to the reduction of cellulose crystallinity that has been observed by several authors [64,65,66]. The amorphization of the cellulose structure is more pronounced in ball-milling in comparison to jet-milling [4], due to repeated impacts of balls on cellulose chain assemblies in crystals [67].

An increase of the water holding capacity (WHC) has been also reported attributed to the increase in specific surface area which leads to the exposure of more hydrophilic groups from cellulose and hemicelluloses [68,69]. However WHC could also decrease with the amorphization of the cellulose with reduction of porosity and it is difficult to predict this evolution in case of ultrafine biomass grinding [70].

## 6. Energy Consumption during Comminution

Several parameters are responsible for the energy consumption during the comminution such as the mechanical properties of the material, the size of the particles before and after milling, the moisture content, the feed rate and the mill characteristics.

Theoretically, in the case of a perfect single elastic sphere during a Brazilian test, it is possible to predict the energy of fracture depending of its tensile strength, Young modulus and Poisson’s ratio [71]. This tensile strength needs to be determined experimentally and is varying with the composition of the material but also with its microstructure which is changing along with downsizing. As Weibull [72] and Epstein [73] have already explained, the number and size of defects in the material decrease with the size of the particles which leads to an increase of its resistance. This has been observed for single particle fracture with different rocks and minerals [74,75]. This effect can be particularly strong for lignocellulosic materials as they exhibit a complex and heterogeneous structure.

The resistance of a particle is also changing with the nature of the load and its application rate. In single particle fracture, a slow compression or a single or double impact configuration do not need the same amount of energy to break the particle. A higher stressing rate can either decrease the strength of some materials by the apparition of interferences between elastic waves leading to an early crack propagation in particular in case of visco-plastic materials like lignocellulosic materials [76,77].

In a bed of particles, particle breakage is controlled by the energy intensity but also by the relative position of particles compared to the direction of the force and by the number of contact between particles [78]. The number of contacts between fine and coarse particles increase with the proportion of fine particles. Some coarse particles can then be in an isostatic stress field and do not break under a compression load. If the energy still increases, it is the finest particles that break. As a consequence, a wide particle size distribution can slow down the comminution and can decrease the milling efficiency.

The attraction forces between particles (Van der Waals, electrostatic, capillary forces) also play a major role in energy consumption as they counteract the breaking process in promoting sometimes agglomeration phenomena. These forces are increasing as the particle sizes decrease [29]. Moreover, as the specific surface area is increasing, the friction between particles also increases leading to more energy loss by heat dissipation [79].

Moisture also strongly affect the energy consumption of biomass grinding as can be seen in several studies on various material milling (wood chips, pellets, miscanthus, switchgrass, various seeds, etc.) [69,80,81,82,83]. Increasing the moisture content increases the energy needed to mill the product. This effect can be explained by the fact that moisture increases the shear resistance of the material [56]. In addition, a dried material is leaving voids and cavities reducing the strength of the material facilitating thereby its milling [84].

There are also other sources of energy consumption related to the type and the size of the mill friction of the mechanical parts with the product, the milling media and the air, air flows, instrumentation, over-dimensioned device, etc.

The comparison of milling equipment on the basis of the energy efficiency is difficult to realize as there is no consensus on the definition of the energy efficiency. It is often computed as the ratio of the energy needed to reach a targeted size and the energy effectively used by the mill, which can be defined or measured in different ways. If authors are interested by the economic cost, the electrical consumption of the mill is considered. When a better understanding of the mechanical process is sought, the net energy (the energy consumed by the mill during grinding operation minus the void energy consumption when the mill is empty) measured as close as possible to the grinding chamber (measured by a torque meter mounted on the shaft of the mill for example) is often preferred. As example, in the case where the energy needed to create new surfaces is calculated from the energy of surface of the materials, the energy losses will represent a tremendous amount that can reach 96% to 99% of the total energy used by the mill [74,85]. But if it is computed from the energy needed for breaking single particles, greater efficiencies depending of the mill considered are observed (up to 80% for roller crushers [79]).

## 7. Conclusions

The milling and especially ultra-fine milling of biomass raises a growing interest in many sectors as it can influence the downstream operations and the yield of conversion in bioenergy. Through this literature review, we seek to underline that a good knowledge of the plant raw materials and process parameters can allow to modulate the powder properties. Such work opens the door to a real reverse engineering applied to the production of biomass particles.

We also underline that the factors influencing the energy consumption during the milling are numerous and difficult to predict from the mechanical properties of the raw materials. However, it is clearly stated that the energy consumption increases significantly as particle size decreases. In a perspective of sustainable technologies, it is important to adjust the target particle size to the intended application. For some applications with high-added value as building blocks for polymers and materials or molecule extraction for green chemistry, a higher energy expenditure for milling can be afforded whereas for biomass conversion into energy, it must be kept as low as possible to remain cost-effective.

Thus, the choice a grinder over another remains difficult as it has a direct impact on the ground powder and on energy consumption. Therefore, it must be based on a good knowledge of the technology and of the raw materials, and must integrate numerous constraints. In the following part of this review, we will seek to highlight the main elements to keep in mind for selecting a milling technology and evoke some approaches of process improvement through thermal pretreatment of starting raw materials.

## Figures and Tables

**Table 1:** Composition of different lignocellulosic biomasses, on a dry basis.

Biomass	Cellulose (%)	Hemicellulose (%)	Lignin (%)	Water–Soluble (%)	Ash (%)	References
Alfa	44–47	22–30	12–20	≈4	≈2	[20,21]
Eucalyptus	53–58	17–20	19–22	1–5	<1	[22]
Miscanthus	43–50	24–34	9–12	1–2	2–4	[23]
Wheat Straw	33–40	21–26	11–23	4–10	7–10	[24,25]
Wood (Pine)	45–50	20–30	25–30	2–10	<1	[26]
Flax fiber	78–80	6–13	2–5	2–4	1–2	[27,28]
Flax shives	32–53	13–21	23–25	1–2	2–3	[28,29,30]
Hemp fiber	67–76	8–12	2–5	2–16	<1	[27,31,32]
Hemp Hurds	39–49	16–23	16–23	0–2	2–4	[33,34,35]
